# A non-randomized concurrent controlled trial of myofunctional treatment in the mixed dentition children with functional mouth breathing assessed by cephalometric radiographs and study models

**DOI:** 10.1186/s12887-022-03559-w

**Published:** 2022-08-25

**Authors:** Janvier Habumugisha, Bo Cheng, Shu-Yu Ma, Min-Yue Zhao, Wen-Qing Bu, Gao-Li Wang, Qiong Liu, Rui Zou, Fei Wang

**Affiliations:** Key Laboratory of Shaanxi Province for Craniofacial Precision Medicine Research, Clinical Research Center of Shaanxi Province for Dental and Maxillofacial Diseases, Department of Orthodontics, College of Stomatology, Xi’an Jiaotong University, 98 Xi Wu Road, Xi’an, Shaanxi 710004 People’s Republic of China

**Keywords:** Mouth breathing, Myofunctional treatment, Cephalometric radiographs

## Abstract

**Objectives:**

This study aimed to examine the clinical effects of myofunctional treatment on children with functional mouth breathing by cephalometric radiographs and study models.

**Methods:**

A total of 224 children (6–10 years old; 114 males and 110 females; SNA°: 82.24 ± 1.67°; ANB°: 2.79 ± 0.80°, 28° < SN-GoGn° < 37°) formed three groups: MB-M group (mouth breathers with myofunctional treatment,*n* = 75); MB-N group (mouth breathers with no treatment,*n* = 70); NB group (nasal breathers with no treatment, *n* = 79). A blind evaluation of cephalometric radiographs and study models was conducted at T1(pre-study) and T2 (post-study), respectively.

**Results:**

Two hundred four children (MB-M:66, MB-N:68, NB:70) completed the present study. At T1, MB-M and MB-N groups, compared to their NB counterpart, had greater anterior lower facial height(*P* < 0.01) and overjet(*P* < 0.001) but shorter overbite and maxillary canines width (*P* < 0.001). At T2, the MB-N group exhibited a higher ANB angle, anterior lower facial height, and overjet, but shorter overbite and maxillary canines width (*P* < 0.001). From T1 to T2, the anterior lower facial height increased, overbite and the maxillary canines width further decreased in the MB-N group (*P* < 0.001). However, in the MB-M group, the incisors were retracted, overbite increased (*P* < 0.001), anterior lower facial height increased insignificantly (*P* > 0.05), and maxillary canines width increased slightly (*P* < 0.05). In the NB and MB-M groups, the mandible showed a normal tendency to grow forward, whereas, in the MB-N group, the mandible showed a tendency to grow downward (*P* < 0.001).

**Conclusions:**

Mouth breathers demonstrated increased anterior facial height and overjet but reduced overbite and maxillary arch width, which improved significantly following myofunctional treatment.

**Trial registration:**

TCTR: TCTR20220401001. Registered 1^st^April 2022-Retrospectively registered.

**Supplementary Information:**

The online version contains supplementary material available at 10.1186/s12887-022-03559-w.

## Introduction

### Background and objective

Mouth breathing occurs when a patient substitutes nasal breathing with a pattern of oral or mixed breathing for more than 6 months [1, 2]. Mouth breathing has a complex etiology that may range from anatomic obstructions such as palatine and pharyngeal tonsil hypertrophy, septal deviation, nasal polyps, nasal turbinate hypertrophy, and allergic rhinitis to harmful oral habits [3–5].

Mouth breathers with no obstructive etiological factors are called functional mouth breathers [6, 7]. Functional mouth breathing is a harmful habit that may interfere with proper craniofacial development.

Mouth breathing jeopardizes maxillofacial muscle functioning and the upper and lower jaws, resulting in abnormal maxillofacial morphology and poor academic performance in children [8, 9]. As a result, proper interventions for mouth-breathing children are required. Before orthodontic treatment, those with nasal obstruction and upper respiratory infection should be treated as soon as possible [1]. It has been reported that adenotonsillectomy enhanced the myofunctional activity and nasopharyngeal airway for most mouth breathers with adenotonsillar hypertrophy [2].

Muscle weakness of the lip and tongue is one of the clinical manifestations of mouth breathers, which leads to abnormal craniofacial development [10, 11]. Kondo Etsuko reported that muscle training positively influenced the management of the different malocclusions and was crucial at the retention stage following orthodontic treatment [12]. Saccomanno et al. proposed that combining orthodontic therapy with functional muscle training might optimize orthodontic treatment stability in individuals with poor oral habits [13].

Oral Myofunctional Therapy (OMT) was described as the “therapy of dysfunctions of the muscles of the face and mouth to improve orofacial functions such as chewing and swallowing and encouraging nasal breathing [14]”. Dr. Farrell created pre-orthodontic trainers (Myobrace System appliances) to increase orofacial muscle training in the early 1990s”. These myofunctional therapies might aid in the correction of children’s tongue posture, swallowing habits, and mouth breathing [15, 16]. Furthermore, the functional orthodontic appliances significantly improved temporomandibular joint disorders (TMD) symptoms in individuals with juvenile idiopathic arthritis and TMD [17].

Although the effectiveness of myofunctional therapy has been questioned, some evidence has been published demonstrating the influence of myofunctional therapy on some dentoskeletal problems [18–20]. Investigating myofunctional treatment in children with functional mouth breathing may lead to a better understanding of myofunctional therapy’s clinical efficacy in individuals with dentofacial abnormalities induced by mouth breathing. It may give valuable information for orthodontic diagnosis and treatment plans in the clinical setting. Hence, this study aims to examine the clinical effects of myofunctional treatment on children with functional mouth breathing by cephalometric radiographs and study models.

## Method

### Trial design

This was a non-randomized concurrent controlled trial involving children who attended the orthodontic clinic of the Stomatological Hospital of Xi’an Jiaotong University, China. This study was carried out following the Helsinki Declaration on medical protocol and ethics, and it was approved by the Medical Ethics Committee of the Hospital of Stomatology, Xi’an Jiaotong University, Registration number: Xjkqll [2018] No.17.

### Participants

#### Eligibility criteria for participants

This study involved 224 young patients from the Orthodontic Department, Stomatological Hospital of Xi’an Jiaotong University. Inclusion criteria: subjects aged 5–10 years; normal body mass index subjects: 18.5<BMI<24.9 [21, 22], Class I molar relationship; Skeletal Class I: ANB°:1–4°, SNA°: 79–85° and normal vertical facial growth: 28° < SN-GoGn° < 37°. Exclusion criteria: subjects with confirmed syndromes and neurologic disorders; subjects who previously received orthodontic therapies, and subjects diagnosed with the following conditions: temporomandibular joint disorders; hypotonia or hyperactivity of the jaw muscles; sleep-disordered breathing (SDB); allergy problems; tongue-tie problems; adenotonsillar hyperplasia, turbinate hyperplasia.

The subjects of this study were grouped into three groups: the MB-M group (functional mouth breathers with myofunctional treatment, *n* = 75) and the MB-N group (functional mouth breathers with no treatment, *n* = 70). The third group was the NB group (nasal breathers with no treatment, *n* = 79). Baseline descriptive data of the three groups were used to check whether the three groups matched age and craniofacial measurements Table 1.

**Table 1:** Baseline demographics describing age and sex for MB-M, MB-N, and NB

	MB-M ***n*** = 75	MB-N ***n*** = 70	NB ***n*** = 79	Total 224	***p***-value
**Sex,** ***n***					
**male**	37(49.3%)	38(54.3%)	39(49.4%)	114(50.9%)	0.791^*a*^
**female**	38(50.7%)	32(45.7%)	40(50.6%)	110(49.1%)	
**Age (y)**	7.41 ± 1.21	7.30 ± 1.21	7.25 ± 1.05	7.32 ± 1.15	0.676^*b*^
**SNA(°)**	82.28 ± 1.64	82.13 ± 1.67	82.29 ± 1.69	82.24 ± 1.67	0.801^*b*^
**ANB (°)**	2.88 ± 0.64	2.59 ± 1.04	2.87 ± 0.65	2.79 ± 0.80	0.061^*b*^

All data are listed as means and standard deviations

SNA (°), the anteroposterior position of the maxilla relative to the anterior cranial base; ANB (°), the relative position between maxilla and mandible

*MB-N* mouth breathers with no treatment, *MB-M* mouth breathers with myofunctional treatment, *NB* nasal breathers

^a^Chi-square test

^b^Oneway Anova

The functional mouth breathers in MB-M and MB-N groups had no upper airways obstructive etiological factors. The absence of upper airway obstruction was established preliminarily by the findings of the otolaryngologist consultations, and habitual mouth breathing was confirmed by clinical history taken from the child’s parents describing the child’s sleeping behavior, such as sleeping with mouth open. An experienced otolaryngologist checked all individuals and confirmed that they were habitual mouth breathers. A complete evaluation by an otolaryngologist comprised a nasopharyngeal x-ray, rhinoscopy, and flexible nasopharyngoscopy; no blockage of the upper airway was observed in all mouth breathing participants. The children in the MB-N and MB-M groups were informed of clinical intervention in the same way by the orthodontist. Still, those in the MB-N group refused any treatments, so orthodontists advised the regular visits. Those in the MB-M group accepted clinical intervention (myofunctional treatment). Although the children in the NB group breathed through their noses, clinical and X-ray tests revealed mild malocclusions; thus, orthodontists also recommended regular visits.

The patients’ baseline characteristics are described in Table 1. The age, ANB°, and SNA° were analyzed with Oneway Anova. The sexes were analyzed with a Chi-square test (α = 5%). There were no statistical differences in the average age, sex, ANB°, and SNA° among the three groups (*P* > 0.05).

#### Interventions

All participants were given the information sheet about our study’s purpose and methods to read, and every child’s parent/guardian signed informed consent to participate.

**Fig. 1:**
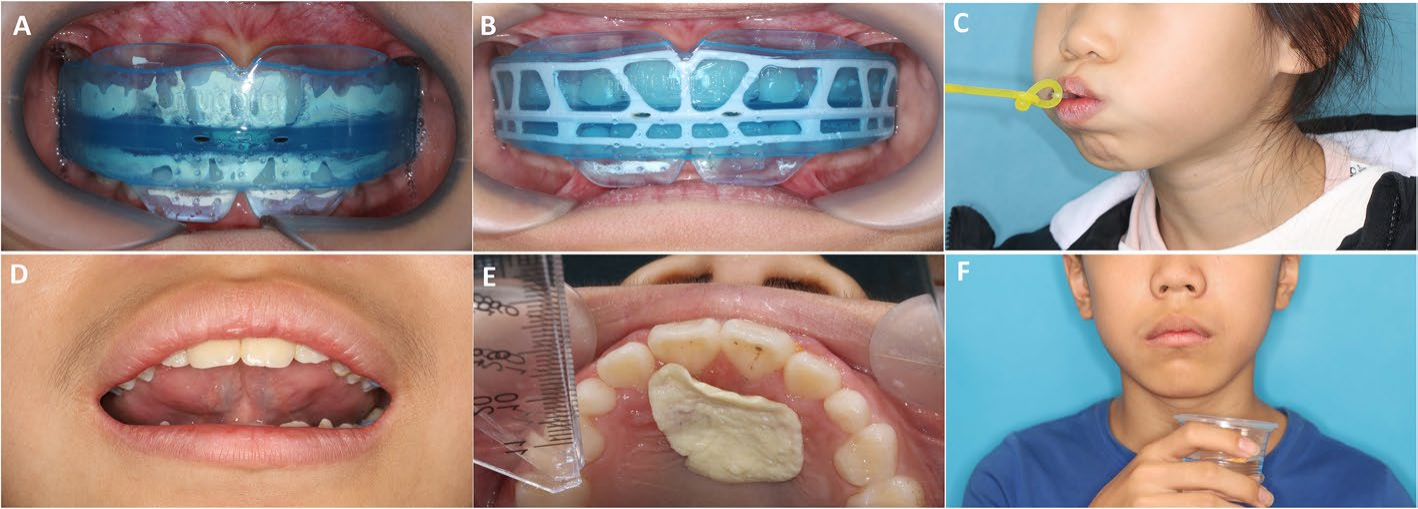
Myofunctional treatment combined with myobrace for kids’ series with oral muscles trainings. **A** MRC-I stage; **B** MRC-II stage; **C** lip sealing training with lip trainer; **D** tongue flipping training; **E** chewing gum training; **F** swallowing training

The children in the MB-M group received orofacial muscle training from one experienced orthodontic nurse: lip sealing training with a lip trainer that tension was 250 g for10 minutes, three times per day; tongue flipping training(the tip of the tongue bouncing at the palate strongly), 100 times per day; chewing gum training (spreading out chewing gum at the palate),15 times per day; swallowing training (pushing 15 ml water on the tip of their tongue up against the hard palate and swallowing with lips closed), 15 times per day. Parents were required to help their children fill out the daily training books. Moreover, children in the MB-M group were asked to wear Myobrace (Myofunctional Research Co. Queensland, Australia) (Fig. 1). The children were instructed to use the trainer by single orthodontist. Children were asked to wear the trainer every day for 2 h during the daytime and night while sleeping. Initial checks were conducted after 2 weeks, with subsequent checks every 4 weeks. The treatment process for MB-M children was 1–1.5 years when the severity of mouthing breathing was decreased. Children in the MB-N and NB groups were advised the regular visits by orthodontists over the same period.

### Outcome measures

#### Cephalometric analysis

**Fig. 2:**
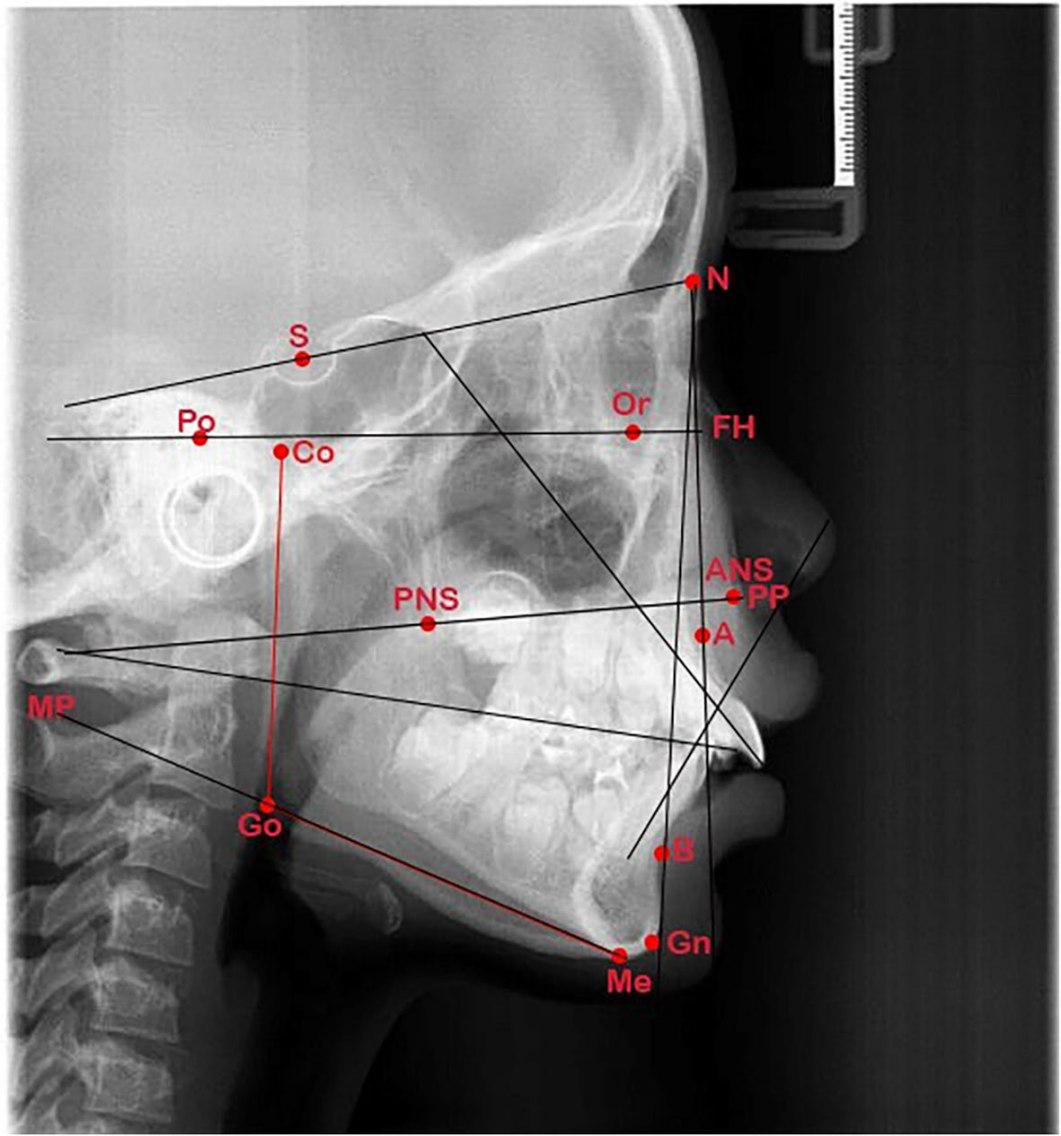
Landmarks of Cephalometric X-ray. Abbreviations of fig. 2: PNS: Posterior nasal spine, ANS: Anterior nasal spine, S: Sella, N: nasion, A: A-point, B: B-point, GO: Gonion, GN: Gnathion, Me: Menton, Po: Porion, Or: Orbitale, CO: condylion FH: Frankfort horizontal plane, PP: Palatal plane, MP: Mandibular plane, OP: Occlusal

The cephalometric radiographs were analyzed by a calibrated investigator with Dolphin 11.5 (Dolphin Imaging and Management Solutions, Chatsworth, Calif). The investigator was blinded to the information of patients. The landmarks of cephalometric radiographs are shown in Fig. 2.

#### Study model analysis

**Fig. 3:**
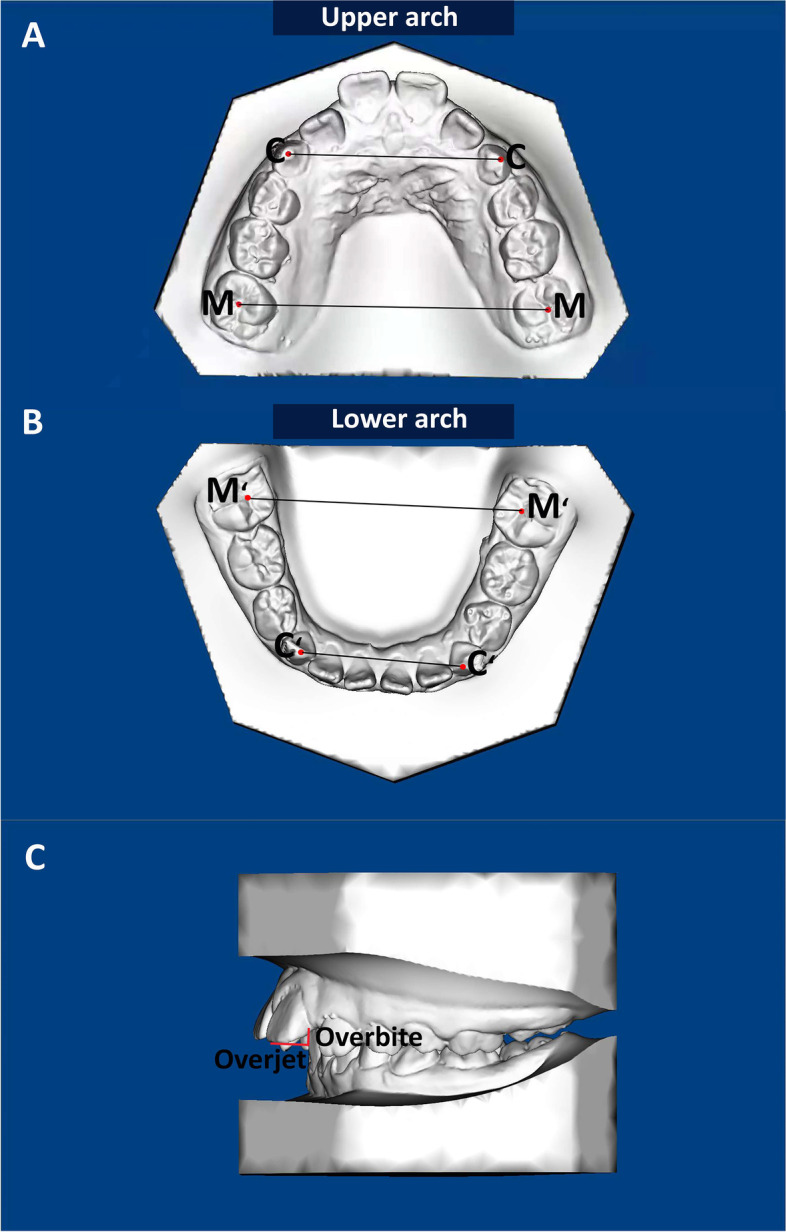
Model measurements. **A**: CC: Canines width of maxilla; MM: Molars width of maxilla; **B**: C′C′: Canines width of mandible; M’M’: Molars width of mandible; **C**: Overjet and overbite measurements

The study models and cephalometric radiographs were collected at T1 and T2(after 13.0 ± 1.1 months). Study models were blocked to avoid the risk of bias, and the landmark of the study models are shown in Fig. 3. The study models were measured with digital calipers (Tesa Technology, Renens, Switzerland; resolution 0.01 mm).

### Sample size

The sample size was calculated using a formula proposed by Suresh KP [23]. The average standard deviation of 2.9 mm and the mean difference of 1.47 mm of overjet between the mouth and nasal breathing children were adopted from previous research by Harari et al. [24].

$$n=\frac{{\left(r+1\right)\left(\mathrm{Z}\upalpha /2+Z1-\beta \right)}^2{\left(\upsigma \right)}^2}{r\ast {(d)}^{2}}$$

*n* is the sample size; *Zα* is the normal deviate at a level of significance (*Zα* is 1.96 for 5% level of significance and 2.58 for 1% level of significance), and *Z1-β* is the normal deviate at 1-β% power with β% of type II error (0.84 at 80% power and 1.28 at 90% statistical power). *r* = n1/n2 is the Ratio of the sample size required for groups, *r* = 1 (equal sample size); or *r* = 0.5(unequal sample size).*σ* is the pooled standard deviation, and d is the difference of means between groups. Researchers can obtain these values (*σ,* d) from prior research with comparable hypotheses or by performing a pilot study [23]. In the present study, those values (*σ,* d) were adopted from a previous study by Harari et al. [24].

### Statistical method

All cephalometric and study model measurements were repeated for 30 randomly chosen participants at three-week intervals to verify reliability using the intra-class correlation coefficient (ICC). The error was calculated according to Dahlberg [25].

$$SE=\sqrt{\sum \frac{D^2}{2N}}$$

The data were processed with SPSS19.0 (IBM Corp, Armonk, NY). All variables pre-and post-study and intergroup variables were analyzed with paired t-test and Oneway Anova, respectively. All data were collected and processed by the same orthodontist.

## Results

9 (12%) children in the MB-M group who did not do the muscle training or wear myobraces; 2 (2.8%) in the MB-N group, and 9 (11.4%) in the NB group lost to follow-up. The final data of these 20 patients were not analyzed at T2

Our study lasted for 13.0 ± 1.1 months, starting in September 2018 and ending in August 2020. Depending on our research power, the study was terminated once a sufficient sample size was obtained, with 66 children in the MB-M group, 68 in the MB-N group, and 70 in the NB group.

The intraclass correlation coefficient (ICC) ranged between 0.90–0.95 for landmark identification of cephalometric radiographs and study models, confirming the reproducibility and reliability of the method. According to all repeated analyses, the method error was negligible (less than 0.5 mm for linear measurements and less than 0.33° for angular measurements).

The average age was 7.41 ± 1.21 years for the MB-M group, 7.30 ± 1.21 years for the MB-N group, and 7.25 ± 1.05 years for the NB group Table 1.

Angular, ratio measurement results of cephalometric radiographs and linear measurement results of the intragroup and intergroup analysis were shown in Tables 2 and 3.

**Table 2:** Angular and Ratio measurement results of cephalometric radiographs in MB-M, MB-N, and NB groups at T1、, T2, 、T1-T2

	intragroup comparison									intergroup comparison	
	MB-M (*N* = 66)			MB-N (*N* = 68)			NB (N = 70)			T1	T2
	*T1 (Mean* ± *SD)*	*T2 (Mean* ± *SD)*	*T1-T2 P* ^*c*^	*T1 (Mean* ± *SD)*	*T2 (Mean* ± *SD)*	*T1-T2 P* ^*c*^	*T1 (Mean* ± *SD)*	*T2 (Mean* ± *SD)*	*T1-T2 P* ^*c*^	*P* ^*b*^ *values*	*P* ^*b*^ *values*
SNA(°)	82.30 ± 1.66	82.65 ± 1.47	0.008** ↑	82.10 ± 1.69	82.97 ± 1.26	p ≤ 0.001***↑	82.27 ± 1.68	82.96 ± 1.47	p ≤ 0.001***↑	*0.790*	*0.383*
SNB (°)	79.38 ± 1.80	80.05 ± 1.64	p ≤ 0.001***↑	79.53 ± 1.87	79.76 ± 1.37	*0.370*	79.37 ± 1.87	80.40 ± 1.53	p ≤ 0.001***↑	*0.923*	MB-N < NB,0.050* MB-N vs MB-M,0.282 NB vs MB-M,0.174
ANB (°)	2.90 ± 0.81	2.61 ± 0.65	0.004**↓	2.61 ± 1.12	3.21 ± 0.73	p ≤ 0.001***↑	2.84 ± 0.72	2.56 ± 0.83	p ≤ 0.001***↓	*0.065*	MB-N > NB, p ≤ 0.001*** MB-N > MB-M, *p* ≤ 0.001*** NB vs MB-M,0.700
SN-GoGn (°)	34.72 ± 1.45	34.76 ± 1.28	*0.876*	34.79 ± 1.36	35.75 ± 1.02	p ≤ 0.001***↑	33.47 ± 1.84	33.76 ± 1.73	*0.105*	NB < MB-N, *p* ≤ 0.001*** NB < MB-M, *p* ≤ 0.001*** MB-N vs MB-M,0.876	NB < MB-M, *p* ≤ 0.001*** MB-M < MB-N, p ≤ 0.001*** NB < MB-N, *p* ≤ 0.001***
FH-MP (°)	30.77 ± 1.50	31.30 ± 3.00	*0.164*	30.97 ± 1.51	32.29 ± 1.09	p ≤ 0.001***↑	30.67 ± 1.55	30.99 ± 1.51	*0.201*	*0.452*	NB < MB-N, p ≤ 0.001*** MB-M < MB-N, *p* ≤ 0.001*** NB vs MB-M,0.362
S-Go /N-Me	0.68 ± 0.03	0.68 ± 0.03	*0.985*	0.69 ± 0.04	0.66 ± 0.04	p ≤ 0.001***↓	0.70 ± 0.04	0.70 ± 0.04	*0.181*	NB > MB-N, *p* ≤ 0.001*** NB > MB-M, *p* ≤ 0.001*** MB-N vs MB-M,0.148	MB-N < MB-M, p ≤ 0.001*** MB-M < NB, *p* ≤ 0.001*** MB-N < NB, *p* ≤ 0.001***
ANS-Me/N-Me	0.53 ± 0.03	0.54 ± 0.03	*0.070*	0.54 ± 0.03	0.58 ± 0.03	p ≤ 0.001***	0.52 ± 0.03	0.52 ± 0.03	*0.974*	NB < MB-N,0.038* NB vs MB-M,0.073 MB-N vs MB-M,0.507	MB-M < MB-N, *p* ≤ 0.001*** NB < MB-N, *p* ≤ 0.001*** NB vs MB-M,0.434
U1-NA (°)	34.32 ± 3.72	31.77 ± 3.93	p ≤ 0.001***↓	33.53 ± 2.9	34.10 ± 3.07	0.033 *↑	30.14 ± 3.75	30.19 ± 3.75	*0.741*	NB < MB-N, *p* ≤ 0.001*** NB < MB-M, *p* ≤ 0.001*** MB-N vs MB-M,0.188	NB < MB-M,0.011* MB-M < MB-N,0.008** NB < MB-N, p ≤ 0.001***
L1-NB (°)	29.26 ± 2.22	27.23 ± 1.66	p ≤ 0.001***↓	28.82 ± 1.55	29.77 ± 1.51	0.021 *↑	26.62 ± 2.81	26.48 ± 2.77	*0.625*	NB < MB-N, p ≤ 0.001*** NB < MB-M, p ≤ 0.001*** MB-N vs MB-M,0.262	NB < MB-M, p ≤ 0.001*** MB-M < MB-N, p ≤ 0.001*** NB < MB-N, p ≤ 0.001***

Mean: mean of the difference; SD: standard deviation; SNA (°): anteroposterior position of maxilla relative to the anterior cranial base; SNB (°): anteroposterior position of mandible relative to the anterior cranial base; ANB (°): the relative position between maxilla and mandible; SN-GoGn (°): the steepness of mandibular plan in relation to the cranial bases; FH-MP (°): intersection angle of the Frankfort horizontal plane and the mandibular plane; PP-GoGn (°): intersecting angle the palatal plane with the mandibular plane. Reflects the inclination of the mandible relative to the palatal plane; S-Go /N-Me: the Ratio of posterior facial height to anterior facial height; ANS-Me/N-Me: the Ratio of the lower anterior face height relative to the total anterior face height; U1-NA (°): intersection angle of the long axis of upper central incisors and line of the line of NA;L1-NB (°): intersection angle of the long axis of lower central incisors and the line of NB

^*^p ≤ 0 .05

^**^p ≤ 0.01

^***^p ≤ 0.001

^c^Paired t test

^b^Oneway Anova

**Table 3:** Linear measurement results of cephalometric radiographs and study models in MB-M, MB-N, and NB groups at T1、T2、T1-T2

	intragroup comparison									intergroup comparison	
	MB-M (***N*** = 66)			MB-N (***N*** = 68)			NB (***N*** = 70)			T1	T2
	***T1 (Mean*** ± ***SD)***	***T2 (Mean*** ± ***SD)***	***T1-T2 P*** ^***c***^	***T1 (Mean*** ± ***SD)***	***T2 (Mean*** ± ***SD)***	***T1-T2 P*** ^***c***^	***T1 (Mean*** ± ***SD)***	***T2 (Mean*** ± ***SD)***	***T1-T2*** ***P*** ^***c***^	***P*** ^***b***^ ***values***	***P*** ^***b***^ ***values***
**Corpus length (mm)**	65.72 ± 2.32	65.91 ± 2.39	*0.239*	65.85 ± 2.41	66.06 ± 2.61	*0.566*	65.93 ± 2.45	66.78 ± 2.08	0.014 *↑	*0.686*	*0.083*
**N-Me (mm)**	104.41 ± 3.62	106.73 ± 3.64	p ≤ 0.001***↑	104.59 ± 3.62	108.74 ± 3.93	p ≤ 0.001*** ↑	102.77 ± 3.41	104.36 ± 3.30	p ≤ 0.001***↑	NB < MB-N,0.007** NB < MB-M, 0.008** MB-M vs MB-N,0.060	NB < MB-N, *p* ≤ 0.001*** NB < MB-M, *p* ≤ 0.001*** MB-M < MB-N, *p* ≤ 0.001***
**S-Go (mm)**	70.47 ± 2.74	72.03 ± 2.80	p ≤ 0.001***↑	71.56 ± 3.24	71.69 ± 3.36	*0.151*	72.20 ± 3.67	73.10 ± 3.50	p ≤ 0.001***↑	NB > MB-M, 0.008** NB vs MB-N,0.251 MB-N vs MB-M,0.520	NB < MB-N,0.030* MB-N vs MB-M,0.542 NB vs MB-M,0.056
**ANS-Me (mm)**	55.65 ± 2.33	56.30 ± 2.25	p ≤ 0.001***↑	56.12 ± 2.57	59.69 ± 2.50	p ≤ 0.001***↑	53.79 ± 2.69	54.63 ± 2.85	p ≤ 0.001***↑	NB < MB-N, *p* ≤ 0.001*** NB < MB-M, p ≤ 0.001*** MB-N vs MB-M,0.285	NB < MB-M, p ≤ 0.001*** MB-M < MB-N, p ≤ 0.001*** NB < MB-N, p ≤ 0.001***
**Co-Go (mm)**	54.18 ± 2.62	54.48 ± 2.64	p ≤ 0.001***↑	52.63 ± 2.59	53.47 ± 3.04	p ≤ 0.001***↑	52.44 ± 2.47	53.44 ± 3.22	p ≤ 0.001***↑	MB-M > MB-N, *p* ≤ 0.001*** MB-M > NB, p ≤ 0.001*** NB vs MB-N,0.666	0.106
**Ramus height (mm)**	52.30 ± 1.86	52.65 ± 1.97	0.017*↑	52.12 ± 1.57	52.50 ± 1.60	*0.163*	51.92 ± 1.76	52.34 ± 1.85	*0.111*	*0.368*	*0.575*
**U1-NA (mm)**	5.53 ± 0.50	5.24 ± 0.49	p ≤ 0.001***↓	5.62 ± 0.49	5.71 ± 0.53	0.042*↑	5.21 ± 0.57	5.19 ± 0.54	*0.554*	NB < MB-N, *p* ≤ 0.001*** NB < MB-M, *p* ≤ 0.001*** MB-N vs MB-M,0.290	MB-M < MB-N, *p* ≤ 0.001*** NB < MB-N p ≤ 0.001*** NB vs MB-M,0.537
**L1-NB (mm)**	3.98 ± 0.55	3.90 ± 0.49	0.003**↓	3.99 ± 0.77	4.23 ± 0.74	0.030*↑	3.73 ± 0.48	3.72 ± 0.48	*0.832*	NB < MB-N,0.015** NB < MB-M,0.017** MB-N vs MB-M,0.936	MB-M < MB-N, *p* ≤ 0.001*** NB < MB-N, *p* ≤ 0.001*** NB vs MB-M,0.076
**Overjet (mm)**	3.74 ± 0.83	3.03 ± 0.66	p ≤ 0.001***↓	3.67 ± 0.85	4.34 ± 0.91	p ≤ 0.001***↑	3.35 ± 0.57	3.18 ± 0.48	0.013*↓	NB < MB-N,0.004** NB < MB-M,0.003** MB-N vs MB-M,0.620	MB-N > NB, *p* ≤ 0.001*** MB-N > MB-M, p ≤ 0.001*** NB vs MB-M,0.222
**Overbite (mm)**	−1.70 ± 1.10	0.52 ± 1.06	p ≤ 0.001***↑	−0.63 ± 0.98	−1.243 ± 0.99	p ≤ 0.001***↓	0.00 ± 1.35	0.04 ± 1.28	*0.616*	MB-N < NB, *p* ≤ 0.001*** MB-M < MB-N, *p* ≤ 0.001*** MB-M < NB, *p* ≤ 0.001***	NB < MB-M, p ≤ 0.001*** MB-N < MB-M, p ≤ 0.001*** MB-N < NB, p ≤ 0.001***
**C-C (mm)**	28.82 ± 1.45	29.90 ± 1.72	0.020*↑	28.91 ± 0.90	28.18 ± 1.08	p ≤ 0.001***↓	29.34 ± 0.93	29.99 ± 0.98	0.042 *↑	MB-N < NB, p ≤ 0.001*** MB-M < NB, p ≤ 0.001*** MB-N vs MB-M,0.612	MB-N < MB-M, p ≤ 0.001*** MB-N < NB, p ≤ 0.001*** NB vs MB-M,0.155
**M-M (mm)**	45.19 ± 2.02	45.26 ± 2.05	*0.071*	45.03 ± 2.08	44.88 ± 2.20	0.014 *↓	45.23 ± 2.17	45.16 ± 2.13	*0.183*	*0.845*	*0.720*
**C′- C′(mm)**	26.84 ± 1.04	26.75 ± 1.05	*0.099*	26.66 ± 1.12	26.67 ± 1.09	*0.812*	26.36 ± 1.45	26.48 ± 1.14	*0.233*	*0.061*	*0.318*
**M’- M’(mm)**	44.75 ± 1.74	44.83 ± 1.70	*0.069*	44.43 ± 1.46	44.55 ± 1.55	*0.122*	44.51 ± 1.31	44.75 ± 1.38	p ≤ 0.001***↑	*0.431*	*0.544*

Ramus height (mm), measured parallel to the tangent at the posterior border of the ramus between the most cranial point of the condyle and the intersection point with the lower border of the ramus mandibulae (the Gonial point (Go)); Corpus length (mm): Length of the mandibular body; N-Me (mm): anterior facial height; S-Go (mm): length from Sella to Gonion; ANS-Me (mm): anteroinferior facial height; Co-Go (mm): Length from Condylion to Pogonion; U1-NA (mm): a horizontal distance from the tip of the maxillary central incisor to the line of NA; L1-NB (mm): a horizontal distance from the tip of mandibular central incisor to the line of NB; IMPA (°): angle between the long axis of lower incisor and mandibular plane; Overjet (mm): horizontal overlap of upper and lower incisors; Overbite (mm): vertical overlap of upper and lower incisors

*MB-N* mouth breathers with no treatment, *MB-M* mouth breathers with myofunctional treatment, *NB* nasal breathers, *T1* pre-study, *T2* post-study

^*^p ≤ 0 .05

^**^p ≤ 0.01

^***^p ≤ 0.001

^b^Oneway Anova

^c^Paired t-test

At T2, compared with the NB group, children in MB-M and MB-N groups had greater N-Me, ANS-Me, SN-GoGn, and ANS-Me, but lower S-Go/N-Me ratio (*P* < 0.001). Compared with the other two groups, children in the MB-N group had greater U1-NA(*P* < 0.01); ANB, FH-MP, and L1-NB angles; wider U1-NA, L1-NB, and overjet linear distances; lower S-Me/N-Me Ratio; shorter overbite and C-C linear distances(*P* < 0.001). The results indicated that ANB angle, anterior lower facial height, the inclination of incisors, and overjet were greater, while overbite and maxillary canine width were less for children in the MB-N group.

From T1 to T2, significant changes were observed in all groups. SNA angle and N-Me, ANS-Me linear distances increased significantly in all three groups (*P* < 0.001). It implied that the maxillary grew forward, and the anterior facial heights increased. However, some different changes are shown below:

There were significant increases in SN-GoGn, FH-MP angles, overjet, and ANS-Me/N-Me ratio in the MB-N group, but a significant decrease in S-Go/N-Me ratio, overbite, and C-C linear distances (*P* < 0.001). U1-NA and L1-NB angles and linear distances slightly increased (*P* < 0.05). SNB angle increased significantly in NB and MB-M groups (*P* < 0.001), but there was no significant difference in the MB-N group. It indicated the anterior lower facial heights increased and overbite decreased more in the MB-N group. Moreover, the widths of the maxillary canines were further decreased, and mandibles showed a downward growth rather than a forward growth.

In the MB-M group, U1-NA, L1-NB angles, and U1-NA, L1-NB linear distances decreased significantly; overjet decreased significantly; overbite increased, and C-C linear distances increased slightly (*P* < 0.05). It indicated the mandibles showed significant forward growth in the MB-M group. The incisors were retracted, overjet decreased, and overbite increased. Moreover, excessive increases of the anterior lower facial height and further decrease of the maxillary canine width were corrected.

## Discussion

### Interpretation

This study found that oral myofunctional treatment benefited children with dental malocclusion caused by functional mouth breathing. Previous research has shown that oral myofunctional therapy improves oral muscle function and eliminates oral behaviors, including thumb sucking, nail biting, tongue thrusting, mouth breathing, and poor tongue and lip posture [26–28]. Oral habits are considered a major etiologic factor of temporomandibular disorders (TMD) because they produce traumatic dental occlusion, which may affect the teeth, masticatory muscles, and temporomandibular joints, producing disturbance of the stomatognathic system’s functional balance [29]. The use of functional orthodontic appliances has been shown to benefit growing children with some TMD-related issues [17].

Research has shown that mouth breathing impairs dentoskeletal development and masticatory function and reduces the degree and duration of vertical occlusal force on the posterior teeth in developing children [30]. According to a previous systematic review and meta-analysis research, the major interventions used to correct musculoskeletal problems in children were nasopharyngeal lymphoid tissue removal, orthodontic appliances, muscle training programs, or combinations of the above [31]. In our study, orofacial muscle training included lip sealing, tongue flipping, chewing gum, and swallowing. Moreover, parents helped their children fill out the daily training books, and children wore myobraces to reinforce oral muscle training. Myobraces were pre-fabricated, removable, flexible appliances designed to stimulate the masticatory and facial muscles by lip sealing, training, and restoring the tongue to its correct position.

According to our study, children in the MB-N group were more likely to have increased anterior lower facial height, overjet, and proclination of upper incisors, which were consistent with some research results [32, 33]. Mouth-breathing children were likely to have an increased ratio of anterior lower facial height to posterior height with the clockwise rotation of the mandible [24, 34]. Some researchers have found that the facial characteristics of mouth breathers were related to altered breathing patterns. The isolated tonsil hypertrophy could lead to forwarding and upward rotation of the mandible. The isolated adenoid hypertrophy could cause the mandible to rotate downward and backward, resulting in a significantly decreased ratio of posterior height to anterior height [35]. There are also reports that mouth breathing was not directly related to facial discrepancies [36]. The research inconsistencies may be associated with the types (obstructive or functional), courses, and severity of mouth breathing.

The current study’s control group was the MB-N group, while the nasal breathing group was the blank control group. Individuals in the first group (MB-M) improved to resemble those with nasal breathing in their craniofacial measurements.

In our study, from T1 to T2, the lower facial height of children in the MB-M group did not increase significantly. The upper anterior teeth were retroclined, the overjet reduced, and the overbite increased. There was a slight increase in the width of maxillary canines, suggesting that myofunctional treatment played a role in controlling the lower facial height and promoting the transversal development of the maxillary arch. After passive myofunctional therapy, Chuang et al. reported changes in craniofacial parameters and life quality for children with sleep apnea. They also found improvements in nasal breathing, mandible linear growth, and airway morphology [18]. Unlike our study, Chuang et al. discovered more vertical growth in the anterior facial height in the treatment group subjects, indicating the mandible clockwise rotation trend [18]. These inconsistent findings might be related to the differences in patient characteristics and methodological techniques between our study and Chuang et al’ study.

Myobrace trainer, as an oral muscular trainer, could promote the lateral development of the dental arch for kids with insufficient lateral development of the maxillary arch [37]. It has also been reported that a myobrace trainer could reduce overjet while increasing facial height for patients with ANB angle > 4° [38]. However, our research found that vertical development was controlled after mouth breathers wore myobrace trainers. The different results were probably caused by subjects with different sagittal and vertical skeletal discrepancies. In the MB-M group, the ANB angle was 1–4°, and the SN-GoGn angle was 28–37°, indicating that myofunctional treatment combined with myobrace trainer might have some control over the vertical development of mouth breathers with normal sagittal and vertical growth.

With a treatment of 13.0 ± 1.1 months, 66 children in the MB-M group had improved lip sealing and nasal breathing, consistent with those reports on the improvement of peripheral muscle functions of children with the myofunctional treatment [39–41].

Myofunctional treatment could improve myoelectric activities of the perioral and masticatory muscles, especially for Angle Class II Division I patients. Atypical swallowing was corrected, bruxism was reduced, and aptitude for nasal breathing improved. A significant reduction of open bite and reduction of ANB angle were observed, along with a significant increase in inter-molar width [39–41]. In our study, myofunctional treatment corrected abnormal positions of tongue and mouth breathing habits and improved lip sealing. Meanwhile, myofunctional treatment inhibited vertical facial growth of mouth breathers with skeletal Class I.

Some researchers compared myobrace trainers with functional appliances, concluding that myobrace trainers induced less significant soft tissue and hard tissue changes than activators for patients with Skeletal Class II between ages 8 to 12. Compared to activators, fewer changes in ANB angle, nasolabial angle, overjet, and facial convexity angle were observed for patients with myobrace trainers [42]. However, in a multicenter, prospective randomized clinical research, myobrace trainers were as effective as Andresen activators in correcting overjet, overbite, sagittal molar relation, and lip sealing for patients aged 6–14 years with large overjet [43]. Moreover, a recent systematic review showed that myofunctional treatment improved snoring and mouth breathing habits [44]. The debate over whether myobrace trainers could treat patients with skeletal disharmonies and upper airway problems should be further explored.

As compliance is a key factor of successful myofunctional treatment, the low success rate in treatment with myobrace trainers was mainly due to poor compliance [43]. Another study found that the overall success rate of both myofunctional appliances (myobrace) and activator appliances was relatively low owing to poor compliance, even though their costs were considered inexpensive [45]. In our research, the myofunctional treatments were executed under parents’ supervision, and parents filled out the training books to ensure high compliance. The treatment would be unsuccessful if children refused to follow doctors’ advice. Myofunctional therapies were shown to be effective for mouth-breathing children in our research. Furthermore, greater outcomes might be obtained if other orthodontic therapy could be administered in addition to the myofunctional therapies.

## Limitation

Due to ethical factors, we could not perform randomized controlled, double-blind clinical trials in our study. The risks of selection bias could not be eliminated, which is the present study’s limitation. Therefore, a randomized clinical controlled study on the efficacy of myofunctional treatments in functional mouth breathing participants is recommended.

## Conclusion

After myofunctional treatment, mouth-breathing children showed better dentofacial growth. The excessive increase of the lower facial height was controlled, and the transverse restriction of the maxillary arch was relieved. Simultaneously, the myofunctional treatment resulted in the retraction of upper incisors, which increased the overbite of anterior incisors. It might be helpful for open-bite correction. The impact of myofunctional treatment on three-dimensional face development in children with functional mouth breathing should be validated using 3D data. Furthermore, the impact of myofunctional therapy on fixed appliance treatment and the long-term stability of myofunctional therapy should be investigated.

## Supplementary Information

**Additional file 1: Supplementary Table 1.** Age distribution data in MB-M,MB-N and NB groups. **Supplementary Table 2.** Sex distribution data in MB-M,MB-N and NB groups. **Supplementary Table 3.** ANB(°) data in MB-M,MB-N and NB groups for T1 and T2. **Supplementary Table 4.** SNA(°) data in MB-M,MB-N and NB groups for T1 and T2. **Supplementary Table 5.** SNB(°) data in MB-M,MB-N and NB groups for T1 and T2. **Supplementary Table 6.** SN-GoGn (°) data in MB-M,MB-N and NB groups for T1 and T2. **Supplementary Table 7.** FH-MP (°) data in MB-M,MB-N and NB groups for T1 and T2. **Supplementary Table 8.** S-Go /N-Me data in MB-M,MB-N and NB groups for T1 and T2. **Supplementary Table 9.** ANS-Me/N-Me data in MB-M,MB-N and NB groups for T1 and T2. **Supplementary Table 10.** U1-NA (°)data in MB-M,MB-N and NB groups for T1 and T2. **Supplementary Table 11.** U1-NA (mm) data in MB-M,MB-N and NB groups for T1 and T2. **Supplementary Table 12.** L1-NB (°)data in MB-M,MB-N and NB groups for T1 and T2. **Supplementary Table 13.** L1-NB (mm) data in MB-M,MB-N and NB groups for T1 and T2. **Supplementary Table 14.** Corpus length (mm) data in MB-M,MB-N and NB groups for T1 and T2. **Supplementary Table 15.** N-Me (mm) data in MB-M,MB-N and NB groups for T1 and T2. **Supplementary Table 16.** S-Go (mm) data in MB-M,MB-N and NB groups for T1 and T2. **Supplementary Table 17.** ANS-Me (mm) data in MB-M,MB-N and NB groups for T1 and T2. **Supplementary Table 18.** Co-Go (mm) data in MB-M,MB-N and NB groups for T1 and T2. **Supplementary Table 19.** Ramus height (mm) data in MB-M,MB-N and NB groups for T1 and T2. **Supplementary Table 20.** Overjet (mm) data in MB-M,MB-N and NB groups for T1 and T2. **Supplementary Table 21.** Overbite (mm) data in MB-M,MB-N and NB groups for T1 and T2. **Supplementary Table 22.** C-C (mm) data in MB-M,MB-N and NB groups for T1 and T2. **Supplementary Table 23.** C′- C′(mm) data in MB-M,MB-N and NB groups for T1 and T2. **Supplementary Table 24.** M-M (mm) data in MB-M,MB-N and NB groups for T1 and T2. **Supplementary Table 25.** M’- M’(mm) data in MB-M,MB-N and NB groups for T1 and T2.

## Data Availability

All data generated or analysed during this study are included in this published article [and its Supplementary information files]
